# Fibroblasts Influence Survival and Therapeutic Response in a 3D Co-Culture Model

**DOI:** 10.1371/journal.pone.0127948

**Published:** 2015-06-08

**Authors:** Meher Majety, Leon P. Pradel, Manuela Gies, Carola H. Ries

**Affiliations:** Discovery Oncology, Roche Innovation Center Penzberg, Pharmaceutical Research and Early Development, Penzberg, Germany; Duke University Medical Center, UNITED STATES

## Abstract

In recent years, evidence has indicated that the tumor microenvironment (TME) plays a significant role in tumor progression. Fibroblasts represent an abundant cell population in the TME and produce several growth factors and cytokines. Fibroblasts generate a suitable niche for tumor cell survival and metastasis under the influence of interactions between fibroblasts and tumor cells. Investigating these interactions requires suitable experimental systems to understand the cross-talk involved. Most *in vitro* experimental systems use 2D cell culture and trans-well assays to study these interactions even though these paradigms poorly represent the tumor, in which direct cell-cell contacts in 3D spaces naturally occur. Investigating these interactions *in vivo* is of limited value due to problems regarding the challenges caused by the species-specificity of many molecules. Thus, it is essential to use *in vitro* models in which human fibroblasts are co-cultured with tumor cells to understand their interactions. Here, we developed a 3D co-culture model that enables direct cell-cell contacts between pancreatic, breast and or lung tumor cells and human fibroblasts/ or tumor-associated fibroblasts (TAFs). We found that co-culturing with fibroblasts/TAFs increases the proliferation in of several types of cancer cells. We also observed that co-culture induces differential expression of soluble factors in a cancer type-specific manner. Treatment with blocking antibodies against selected factors or their receptors resulted in the inhibition of cancer cell proliferation in the co-cultures. Using our co-culture model, we further revealed that TAFs can influence the response to therapeutic agents *in vitro*. We suggest that this model can be reliably used as a tool to investigate the interactions between a tumor and the TME.

## Introduction

The tumor-stroma interaction has been identified as a hallmark of cancer[1]. The role of stromal cells in cancer progression has partially been elucidated, and several processes from growth factor secretion to evading immune response have been attributed to the stromal cells. The ratio of tumor stroma has been shown to serve as an independent prognostic factor for breast cancer patients that indicates a three-fold increased risk of relapse for stroma-rich tumors [2]. Further, stroma-related molecular signatures can be used to predict the resistance of breast cancer to neo-adjuvant chemotherapy [3]. A desmoplastic reaction involving a variety of stromal cell types is often described as a distinct unique characteristic of pancreatic cancer [4]. Similarly, stromal cells have also been implicated in cancer progression and prognosis of lung cancer [5].

Fibroblasts constitute one of the most abundant cell types in the tumor stroma [6]. In normal tissues, fibroblasts play an important role in maintaining homeostasis and in wound healing by producing an array of factors that constitute the extracellular matrix (ECM) and other growth factors and cytokines that are essential for healing [7]. The cross-talk between the tumor cells and stromal fibroblasts in the TME influences to the secretion of an array of growth factors and cytokine/chemokines that, in turn, support tumor cell growth or survival, induce neo-vascularization and generate an immuno-suppressive TME in several cancers [8, 9]. Currently, TAFs appear to play a key role in tumor progression, and provide significant predictive or prognostic value, as well as serve as potential therapeutic targets [10].

To understand the mechanisms underlying the cross-talk between tumor cells and TAFs *in vitro*, a co-culture system in which tumor cells can interact with fibroblasts, similar to the TME *in situ*, is required. Conventionally, trans-well chambers (Boyden chambers) are used for this purpose. Using this approach, cells are separated by a porous membrane through which soluble factors are able to diffuse freely but direct cell-cell interaction is absent. The importance of direct cell-cell contact in this context has been demonstrated by experiments showing that the collagen-based co-culture of breast cancer cells with serum-activated fibroblasts induced clonogenic growth *in vitro* [11]. Recently, it has been shown that the direct interaction between luminal-/ basal-like breast cancer cells and fibroblasts invokes distinct phenotypic and gene expression changes that differ from trans-well co-cultures [12]. In addition, *Fujita et al*., showed that pancreatic cancer cell proliferation was enhanced by directly co-culturing these cells with pancreatic stromal cells, allowing the two cell types to directly interact in the culture dishes [13]. However, these studies were performed by culturing either one of the cell types on a flat 2D surface, which hardly represents the complex TME *in vivo*. It has been clearly demonstrated that the 2D culture system, although convenient for most applications, is a poor environment to study dynamic cellular interactions [14, 15]. Alternatively, 3D culture of cells provides an environment that preserves several phenotypic and functional characteristics of primary cells/tumors that reflects the *in vivo* conditions to a certain but significant extent. This culture system has been described to induce a gene expression pattern that is similar to that under *in vivo* conditions and to influence a response to therapeutic compounds *in vitro* that correlates with and may provide potential predictive value with regard to the clinical response[16, 17].

In the present study, we developed a 3D co-culture system that enables the formation of multi-cellular spheroids in suspension containing direct cell-cell contacts between tumor cells and fibroblasts in serum-free medium. Using this co-culture system, we identified cancer cell lines that depended on co-cultured fibroblasts co-culture for survival in serum-free conditions. Further, we demonstrated that this tumor cell-fibroblast co-culture system influences the response to therapeutic agents in a manner that reflects the clinical situation in patients.

## Materials and Methods

### Antibodies

The antibodies used for the treatment of cells in the cell viability assays were obtained from various sources as follows:—mAb IGF1R (*R1507*) and the cMet antibody (Onartuzumab) were generated in-house as described in the patents *US7572897* and *US7476724*, respectively. Erbitux /Cetuximab was obtained from Merck KGaA, Darmstadt, Germany. The anti- IL6, mAb (#MAB227) was obtained from R&D Systems GmbH, Wiesbaden-Nordenstadt, Germany. For flow cytometry, goat anti-human EpCAM/Trop-1 (# AF960), anti-human FAP antibody (# MAB3715), Isotype control antibodies (#AB-108-C and #MAB002) and the secondary antibodies, APC-labeled antibody for EpCAM (#F0108) and Alexa488-labeled antibody for FAP (#A21202) were purchased from R&D Systems GmbH, Wiesbaden-Nordenstadt, Germany.

For Western blotting, the EGF Receptor (D38B1) XPRabbit mAb (#4267), the phospho-EGF Receptor (Tyr1068) antibody (#2234), the c-Met (L41G3) mouse mAb (#3148), the phospho-c-Met (Tyr1234/1235) (D26) XPRabbit mAb (#3077), the phospho-Stat3 (Tyr705) (D3A7) XPRabbit mAb (#9145), the Stat3 antibody (#9132) and the HRP-labeled anti-rabbit (#7074) and anti-mouse secondary antibodies (#7076) were all obtained from Cell Signaling Technology (New England Biolabs, Frankfurt am Main, Germany). Magic Mark XP (#LC5602, Life Technologies GmbH, Darmstadt, Germany) was used a molecular weight marker for Western blotting. Lumi-Light PLUS (#12015196, Roche Diagnostics Deutschland GmbH, Mannheim, Germany) was used as the HRP substrate for immuno-detection.

### Cell culture

All cell lines were cultured for passaging in cell culture flasks in media containing 10% FBS, 2 mM L-glutamine, 1% penicillin- streptomycin and 1% non-essential amino acids as recommended by the provider. The cells used for further experiments were below passage 15.

### Co-cultures and cell viability assay

Boyden-chamber assays were performed using trans-well plates from (#3391, Corning Incorporated). Three thousand fibroblasts were seeded in the upper chamber with the membrane filter, and 2000 cancer cells were seeded in the bottom chamber. The 2D co-culture was performed in 96-well plates. Five thousand tumor cells were seeded per well for mono-cultures and 2000 tumor cells and 3000 MRC5 fibroblasts per well in 96 well plates for co-cultures. We performed 3D co-cultures in 96 well plates (#655098, Greiner Bio-One, Frickenhausen, Germany) coated with poly-2-hydroxyethyl methacrylate (#18894–100, Polysciences Europe GmbH, Eppelheim, Germany). The tumor cell lines were cultured either as mono-cultures or co-cultures with the MRC5 fibroblast cell line, or with primary tumor-associated fibroblasts (TAFs) for 5 days at 37°C in an incubator containing 5% Co2 in serum-free media supplemented with 5% Panexin NTA lacking hormones and growth factors (#P04-95700, PAN-Biotech GmbH, Aidenbach, Germany), 1% penicillin- streptomycin (#15140–122, Life Technologies GmbH, Darmstadt, Germany), 2mM L-glutamine (#P04-80100, PAN-Biotech GmbH, Aidenbach, Germany) and 1% non-essential amino acids (#11140–035, Life Technologies GmbH, Darmstadt, Germany). Where indicated, the cells were treated with therapeutic antibodies or respective controls from day 0. Cell viability was measured on day 5 using the CellTiterGlo Luminescent cell viability assay (#G7571, Promega, Mannheim, Germany). An Equal volume of CellTiterGlo reagent was added to each well and was mixed by re-suspension. The plates were incubated at room temperature on a shaker for 30 min and re-suspended again. The relative luminescence units (RLU) were measured using a microplate reader (Infinite 200 Pro, Tecan Deutschland GmbH, Crailsheim, Germany).

### Measurement of secreted growth factors/cytokines

Supernatants were collected from the 5-day co-cultures were collected and were either used immediately or were stored at -80°C until further use. The Human cytokine/chemokine 96-well plate assay was used to measure 42 different cytokines in the supernatants (#MPXCYTO60KPMX42-42 Multiplex, Merck Chemicals GmbH, Darmstadt, Germany). Specific analytes that were not included in the 42-plex (#HCYP3MAG-63K–MCSF, #HADCYT-61K-HGF, #HIGF-52K-01-IGF1, #TGFB-64K-03-TGFß, Merck Chemicals GmbH, Darmstadt, Germany)) were purchased and used to measure additional growth factors. This assay was performed according to the manufacturer’s instructions. Briefly, 2.5 x 10^5^ tumor cells or fibroblasts per well were seeded as mono-cultures or for co-cultures 1x10^5^ tumor cells were combined and 1.5 x 10^5^ fibroblasts per well and were seeded as co-cultures in 2 ml of DMEM supplemented with 5% Panexin NTA on polyHEMA polyHEMA-coated 6-well plates as described for the cell viability assay. Undiluted supernatants were incubated with capture beads or a bead mix overnight at 4°C in the provided 96-well filter plates. Then, the beads were washed and incubated with the detection antibody for one hour at RT in the dark, followed by incubation with Phycoerythrin-labeled streptavidin for 30 minutes at RT in the dark. Next, the beads were washed twice, and the mean fluorescence intensity (MFI) was measured using a Bioplex 2000 instrument (#660–0000, Bio-Rad Laboratories GmbH, Munich, Germany). The analysis was performed using the 5-parameter logistic regression tool in Bioplex manager software (version 6.0).

### Microscopy

The cells were cultured as a mono-culture or a co-culture as indicated for the cell viability assay, and images were captured on day 5 using an inverted microscope (Leitz Labovert microscope, Leica microsystems, Wetzlar, Germany) at a 20x magnification. For confocal imaging, the cells were trypsinized and washed once with warm PBS followed by a wash with warm serum-free DMEM. The tumor cells were incubated in 10 μM Cell Tracker Green 5-chloromethylfluorescein diacetate (CMFDA; #C2925, Life Technologies GmbH, Darmstadt, Germany), and the fibroblasts were incubated in 10 μM Cell Tracker Red CMTPX (#C34552, Life Technologies GmbH, Darmstadt, Germany) in serum-free medium for 15 min. Then, the cells were washed twice with warm PBS. The labeled tumor cells (2.5x10^5^) were cultured either alone or in co-culture with the labeled MRC5 fibroblasts (at a 1:1.5 ratio) for 5 days in polyHEMA-coated 6-well plates. On day 5, the spheroids were washed three times with warm PBS and then fixed using 4% PFA in PBS for 20 min at RT. After fixation, the spheroids were washed once with PBS and mounted in mounting medium before imaging. Z-stack sections of the spheroids were captured using a confocal laser scanning microscope (40 x magnifications, Nikon A1 laser scanning microscope, Nikon GmbH, Dusseldorf, Germany).

### Statistical analysis

Data analysis was performed using GraphPad Prism Software version 6.0 (La Jolla, CA, USA). Cell proliferation in the mono-cultures and co-cultures and the responses of the mono-cultures and the co-cultures to treatment with therapeutics agents were compared using two-way ANOVA, followed by posttest analysis using the Holm-Sidak method. P<0.05 was considered to be significant. (The p-values are represented as follows: 0.01–0.05 = *, 0.01–0.001 = **, 0.001–0.0001 = ***, < 0.0001 = ****.)

## Results

### Three dimensional co-culture of cancer cells with fibroblasts induces differential survival

**Fig 1:**
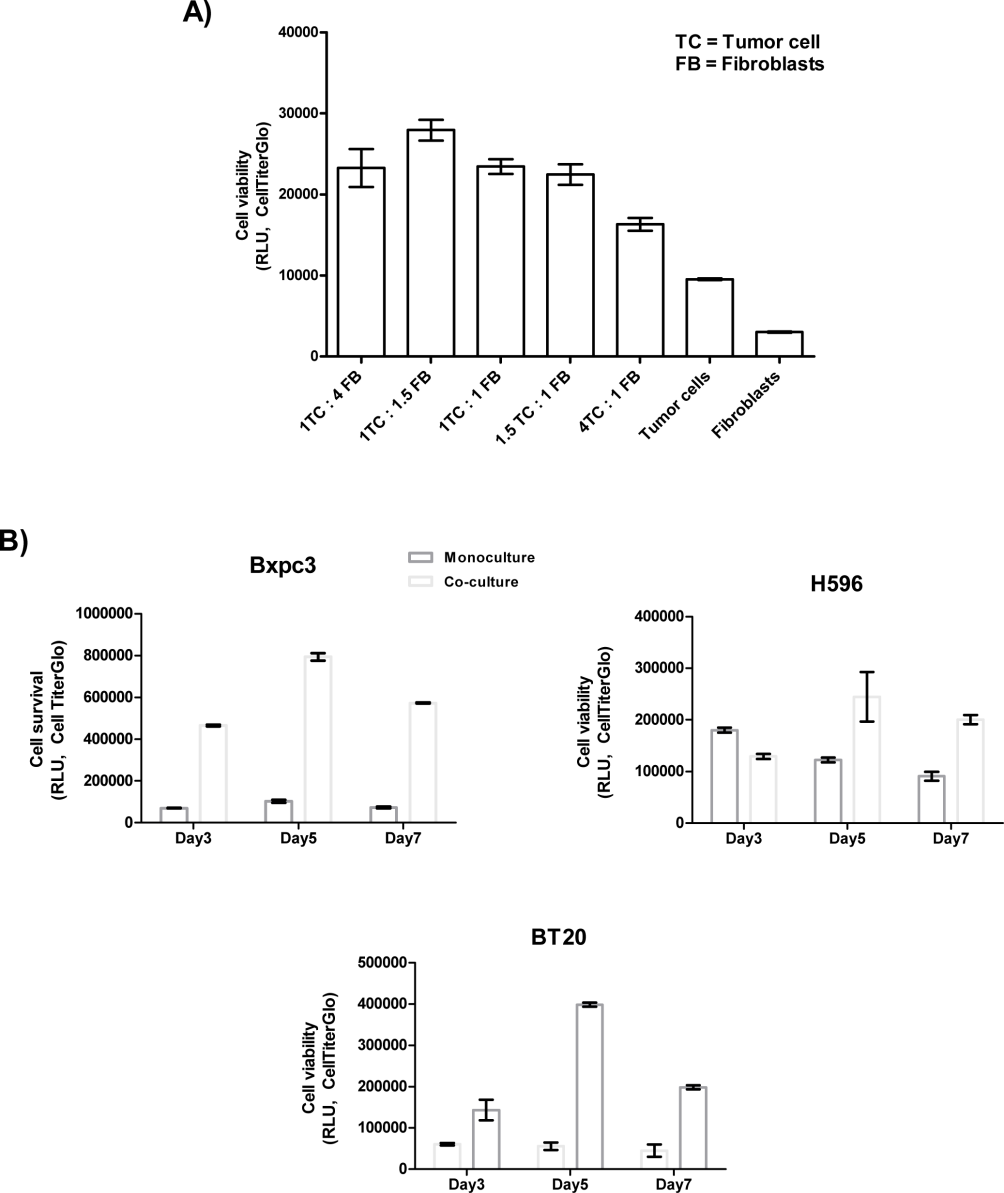
Optimization of co-culture system. **A) Optimization of the tumor cell: fibroblast ratio.** Cancer cells and fibroblasts (MRC5) were cultured in 96 well-well plates as either monocultures or co-cultures as described in the cell viability assay in the Materials and Methods section. Different ratios of tumor cells to fibroblasts were used as indicated. Cell viability was measured on day 5. We observed that the cell viability on day 5 was the highest at tumor cell: fibroblasts ratio of 1:1.5) on day 5. **B) Optimization of the co-culture duration.** Cancer cells and fibroblasts (MRC5) were cultured in 96-well plates as monocultures or co- cultures as described in the cell viability assay in the Materials and Methods section. The tumor cells and fibroblasts were co-cultured at a ratio of (1:1.5), and cell viability was measured on days 3, 5 and 7. The viability in of the co- cultured cells increased from day 3 to day 5 and then decreased slightly on day 7.

We tested different ratios of tumor cells and MRC5 fibroblasts at various time points (from day 3 to day 7) to understand the growth kinetics of the co-cultures. Although increased survival was observed at all of the tested ratios, the ratio of 1 tumor cell to 1.5 MRC5 fibroblasts resulted in the highest cell survival (Fig 1A). We further observed that cell survival values, increased from day 3 to day 5 and then decreased in most of the cell lines by day 7 (Fig 1B). Hence, we selected the 1:1.5 ratio and day 5 as a suitable time point to measure cell survival and cytokine secretion by the co-cultures in the screening experiments.

**Fig 2:**
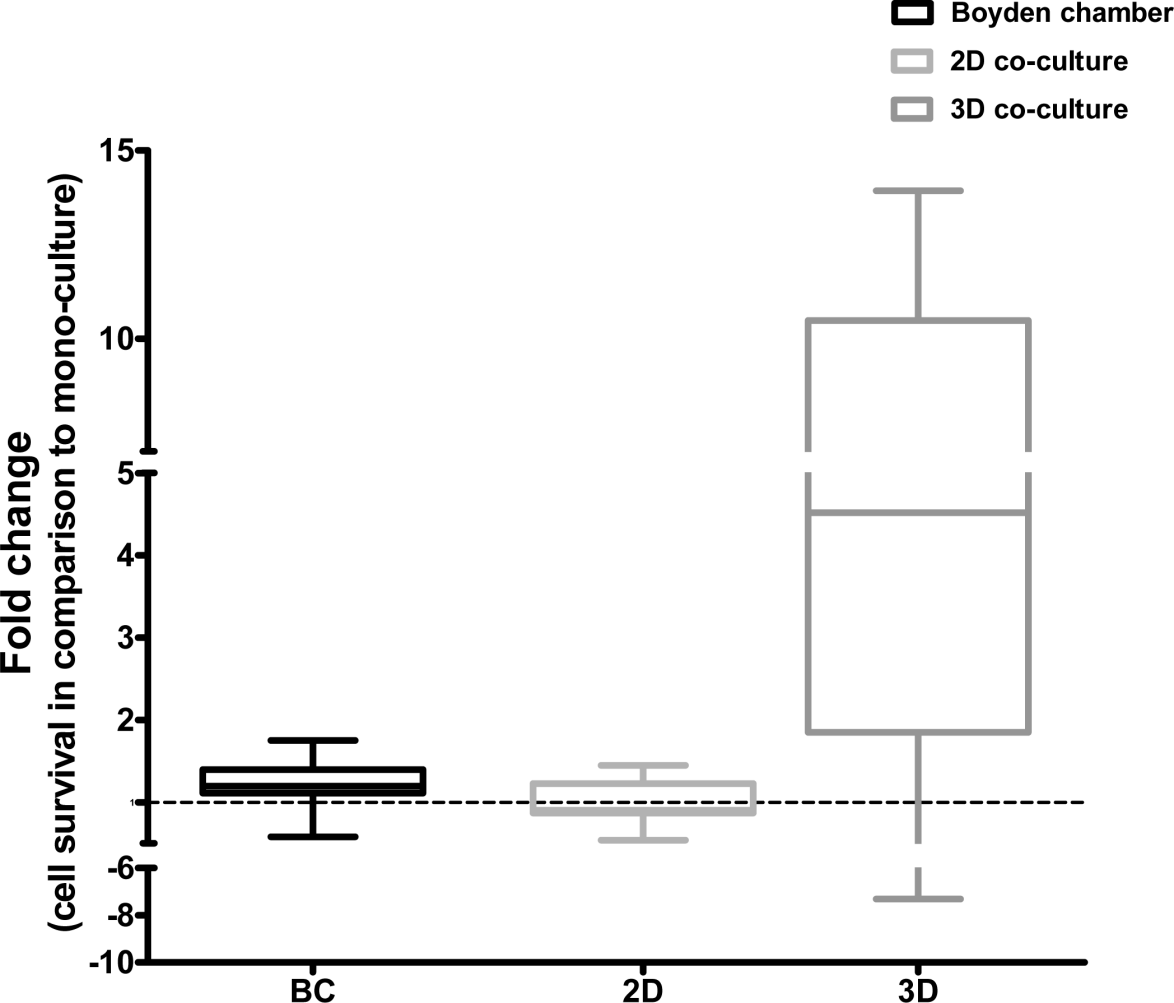
Comparison of the Boyden chamber, 2D co-culture and 3D co-culture systems. To compare the 3D co-culture system to the 2D co-culture and trans- well co-culture systems, tumor cells and fibroblasts were cultured as either as mono-cultures or co-cultures for 5 days as described in the Materials and Methods section. Cell viability was measured on day 5. We observed that 3D co-culture of the tumor cells with fibroblasts induced differential proliferation in co-cultures compared to the Boyden chamber or the 2D co-culture system.

Using these conditions, we then compared the influence of 3D co-cultures on the survival of pancreatic cancer cells with that of 2D and trans-well co-cultures. The results of this comparison indicated that 3D co-culture indeed induced differential cell survival in comparison to 2D co-culture and trans-well co-culture (Fig 2).

### Three dimensional co-culture supports cell survival in a tumor type-specific manner

To determine if the direct 3D co-culture of fibroblasts and tumor cells influences the survival of tumor cells from different indications (Table 1), we co-cultured a panel of pancreatic, lung and breast cancer cells with MRC5 fibroblasts and compared the tumor cell viability between the tumor cell mono-cultures and the co-cultures. For each cancer type, we identified cell lines that exhibited increased survival in co-culture with fibroblasts and other cell lines that did not exhibit this increase in survival.

**Table 1:** Cell line panel.

Catalog #	Tumor cell line	Source
**Lung cancer**		
CCL-185	A549	LGC
CRL-5908	NCI-H1975	LGC
CRL-5909	NCI-H1993	LGC
CRL-5800	NCI-H23	LGC
CRL-5807	NCI-H358	LGC
HTB-177	NCI-H460	LGC
CRL-5810	NCI-H522	LGC
HTB-178	NCI-H596	LGC
**Breast cancer**		
HTB-19	BT20	LGC
HTB-20	BT474	LGC
HTB-22	MCF7	LGC
HTB-26	MDAMB231	LGC
HTB-131	MDAMB453	LGC
HTB-132	MDAMB468	LGC
HTB-30	SKBR3	LGC
	JIMT1	DSMZ
	KPL4	DSMZ
**Pancreatic cancer**		
CRL-1687	BxPc3	LGC
CRL-1469	Panc1	LGC
HTB-79	Capan1	LGC
HTB-80	Capan2	LGC
CRL-2119	HPAC	LGC
CRL-1682	AsPc1	LGC
	PK45P	Oncotherapy Science, Inc.
	Suit2	Oncotherapy Science, Inc.
	PancTu-1	DSMZ
**Fibroblasts**		
PC60161A (primary breast TAFs)	161A	Asterand, PLC.
PC60129A1(primary lung TAFs)	129A	Asterand, PLC.
CCL-171	MRC5	ATCC
SCR013	LT2	Millipore corporation

**Fig 3:**
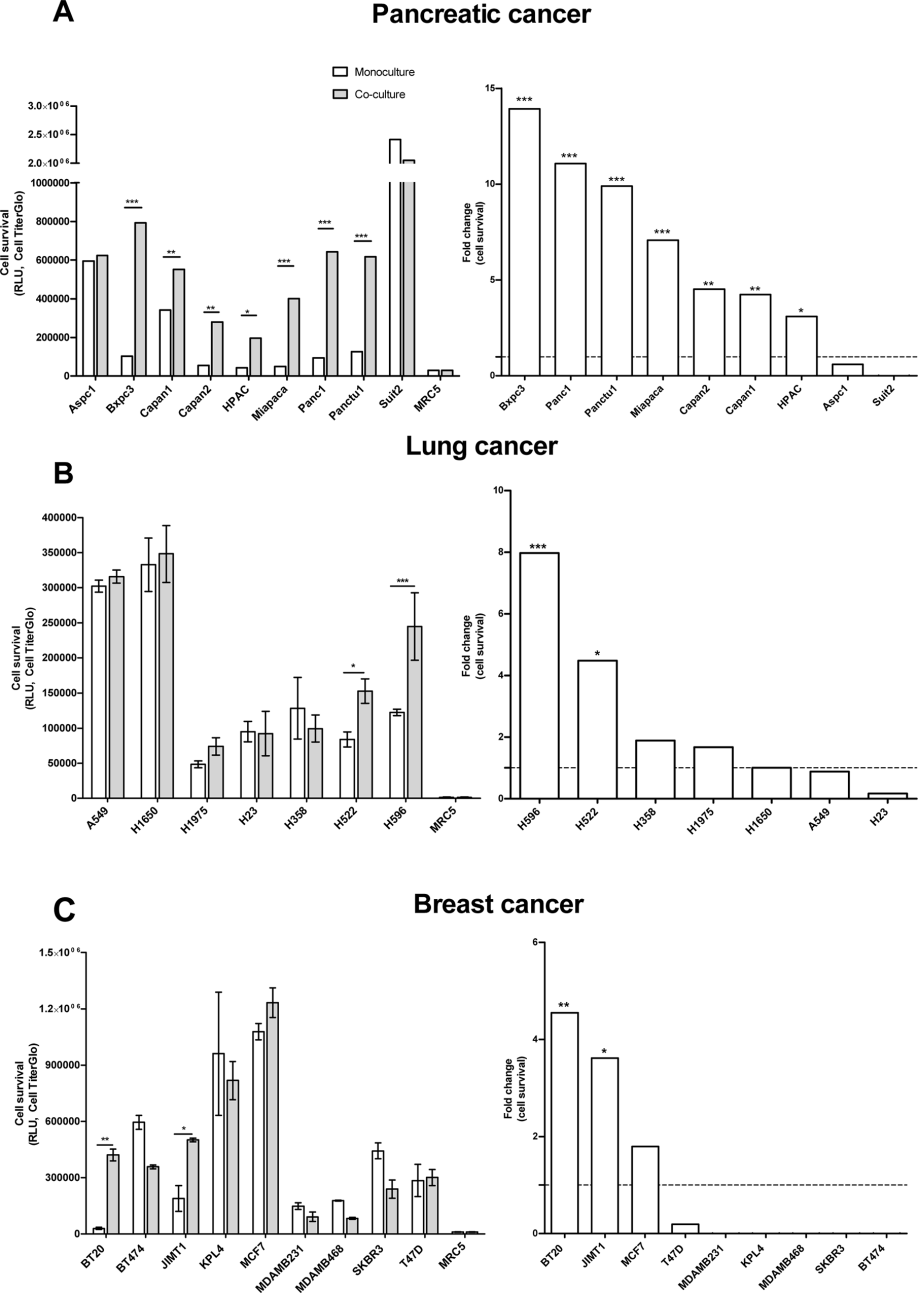
Co- culturing the tumor cells with MRC5 fibroblasts influences cell survival. Tumor cells and MRC5 fibroblasts were cultured as either co- cultures or monocultures as described. Cell viability was measured based on the total ATP content on day 5 after cell seeding using CellTiterGlo. **A)** Seven of the 9 pancreatic cancer cell lines showed exhibited a significant increase in cell survival upon co- culturing with MRC5 cells. Bxpc3 cells exhibited the greatest fold-change in proliferation among these cell lines upon co-culturing. **B)** Two out of the 7 of the lung cancer cell lines exhibited a significant increase in cell survival upon co- culturing with MRC5 cells; out of which the H596 cells exhibited the greatest fold-change in proliferation upon co-culturing. **C.** Of the two breast cancer cell lines that exhibited an increase in proliferation upon co-culturing with MRC5 fibroblasts, only the BT20 cells exhibited a significant increase in cell survival.

In the pancreatic cancer cell panel, 7 out of the 9 cell lines exhibited a significant increase in cell survival when co-cultured with fibroblasts. In the lung and breast cancer panels, only 2 out of the 7 and 9 tested cell lines, respectively, exhibited increased survival when co-cultured with fibroblasts (Fig 3).

**Fig 4:**
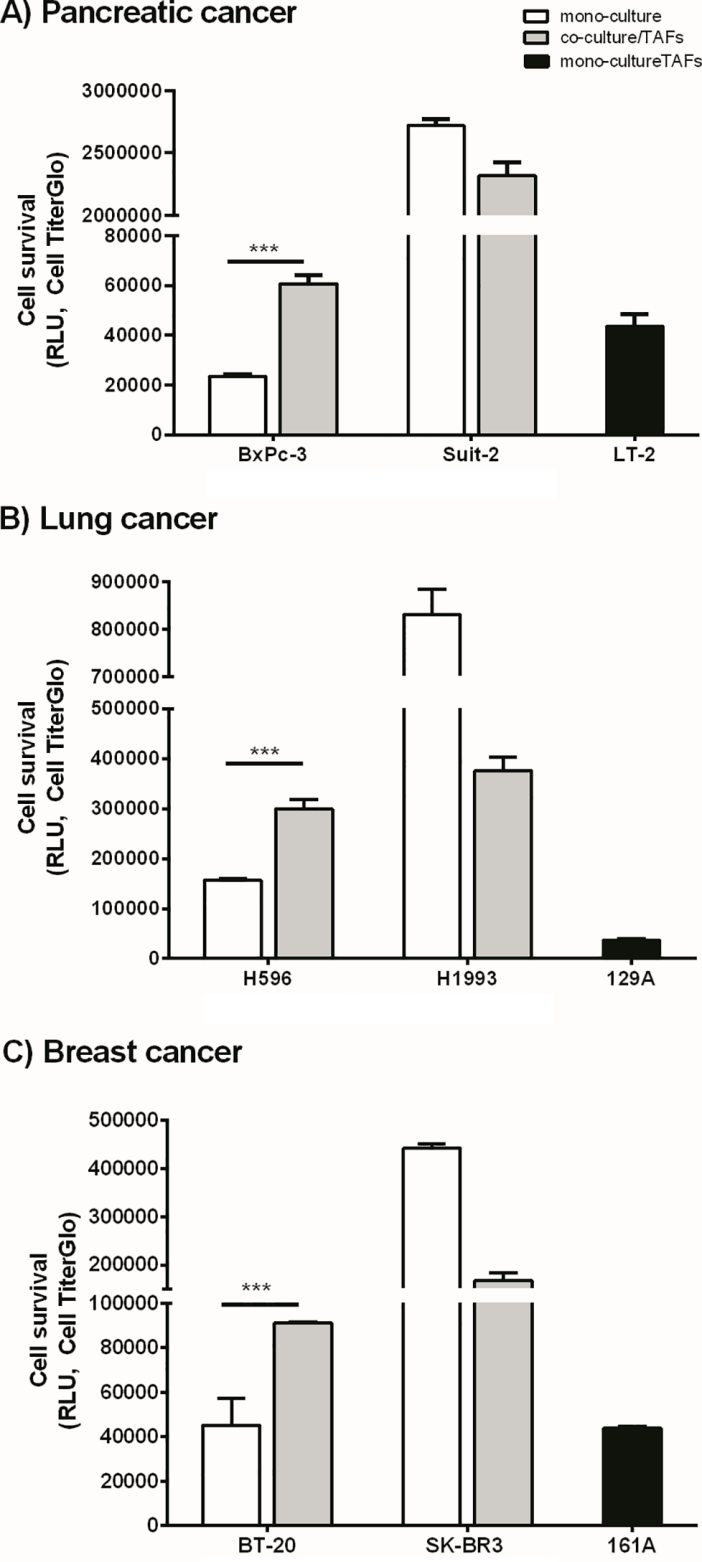
Co- culturing the tumor cells with primary tumor associated fibroblasts (TAFs) influences cell survival similar to MRC5 fibroblasts. One tumor cell line that exhibited the greatest fold-change in cell survival due to co-culturing (Bxpc3, H596 and BT20) and one cell line that did not exhibit an increase in survival upon co-culture with MRC5 cells from each cancer type (Suit2, H1993 and SKBR3) were co-cultured with corresponding primary TAFs (129A, lung TAFs and or 161A, breast TAFs) or organs-specific fibroblasts (LT2, pancreatic fibroblasts) for 5 days followed by measurement of cell viability on day 5 using CellTiterGlo. All three cell lines that exhibited a significant increase in cell survival upon co-culturing with MRC5 fibroblasts (Bxpc3, H596 and BT20) also exhibited increased survival in co-culture with TAFs, whereas the cell lines that did not exhibit increased survival in co-culture with MRC5 (Suit2, H1993 and SKBR3) retained their proliferative properties even upon co-culturing with TAFs.

To validate this observation using primary TAFs, we selected one cell line that exhibited increased survival in co-culture and one that did not exhibit increased survival from each of the cancer panels and co-cultured these cells with organ-specific fibroblasts or primary TAFs. Our data indicated that, similar to the data for the MRC5 fibroblasts, the cell lines that exhibited increased survival in co-culture also exhibited increased survival in the presence of the corresponding primary TAFs or organ-specific fibroblasts (Fig 4).

### Differential secretion of cytokines and growth factors by 3D co-culture spheroids

To investigate the potential role of soluble growth factors and cytokines in the increased proliferation observed in the co-cultures, we analyzed the mono- and co-culture supernatants, on day 5, for the presence of approximately 42 different cytokines. Several cytokines were found to be differentially secreted upon co-culture with MRC5 fibroblasts in the co-culture model (S4 Fig). We focused on the secretion of EGF, HGF and IL6, because these soluble factors have been associated with an important role in tumor progression, resistance and inflammation.

**Fig 5:**
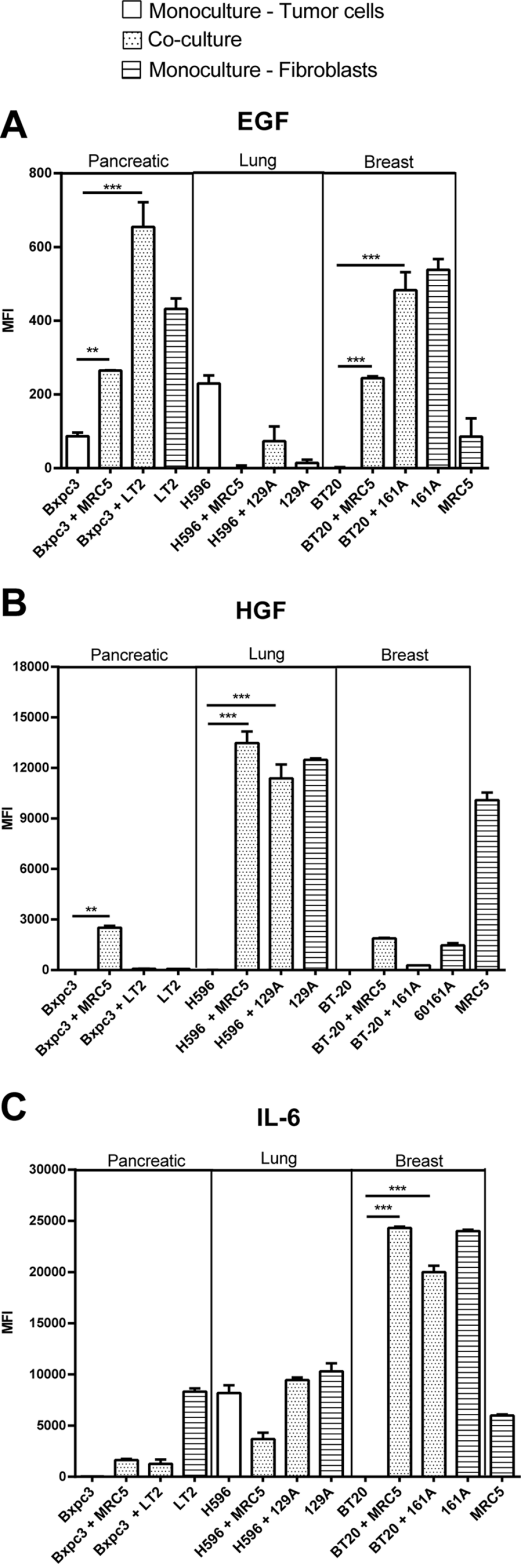
Co- culturing the tumor cells with MRC5 fibroblasts induces differential secretion of growth factors and cytokines. Tumor cells and fibroblasts were co-cultured for 5 days as described, and supernatants were collected. The levels of EGF, HGF and IL6 secreted by the mono- or co-cultures in the supernatants were measured using Luminex multiplex technology. The relative levels of these secreted factors are plotted in the graphs as the mean fluorescence intensities (MFI). The error bars represent the standard deviation of three replicates. **A)** The pancreatic and breast cancer co-culture supernatants contained increased EGF levels compared to the lung cancer co-culture supernatants. **B)** Increased HGF levels were detected in the supernatants from the lung cancer co-cultures but not in those from the corresponding mono-cultures. The Lung fibroblast cell line MRC5 and the TAFs, 129A, also secreted high levels of HGF into the supernatants in monoculture. **C)** High levels of IL6 were detected in the supernatants from BT20 cells co-cultured with MRC5 or 161A primary breast TAFs and in the supernatants from the H596 cell line mono- and co-cultures. The fibroblasts cell lines MRC5, and LT2 and the primary TAFs, 129A also produced IL6 in mono-culture.

EGF was secreted by breast TAFs and pancreatic fibroblast monocultures, by the Bxpc3 cells and BT20 cells in co-culture with fibroblasts (Fig 5A). High level of HGF was detected in the monoculture supernatants from the MRC5 fibroblasts and the primary lung TAFs and in the co-culture supernatant of lung cancer cells (H596) with MRC% fibroblasts or the primary lung TAFs (Fig 5B). IL6 was secreted at high levels by the primary breast TAFs and moderately by the primary lung TAFs and pancreatic fibroblasts. The breast cancer cells (BT20) secreted high levels of IL6 when co-cultured with MRC5 cells and primary TAFs whereas, the monocultures and co-cultures of lung cancer cells (H596) secreted only moderate levels of IL6 (Fig 5C).

Taken together, we observed that EGF was primarily secreted by the co-cultures of pancreatic and breast cancer cells, HGF was secreted by the co-cultures of lung cancer cells and the corresponding fibroblasts and that IL6 was secreted at high levels by the co-cultures of breast cancer cells and the primary breast TAFs.

### Cancer cell-fibroblast co-culture influences the response to therapeutic agents

We further investigated whether the elevated levels of soluble factors in the co-cultures contributed to the increase in cell survival. To this end, cancer cells that were identified to secrete increased levels of the aforementioned soluble factors were allowed to grow as either monocultures or were co-cultured with MRC5 fibroblasts or the corresponding TAFs for 5 days in the presence of inhibitory antibodies against EGFR (Erbitux), mAb cMet (monoclonal antibody cMet), mAb IL6, mAb IGF1R (R1507). Cell survival was measured using CellTiterGlo. The percentage of surviving cells (% survival) was calculated for each treatment relative to the corresponding isotype controls.

**Fig 6:**
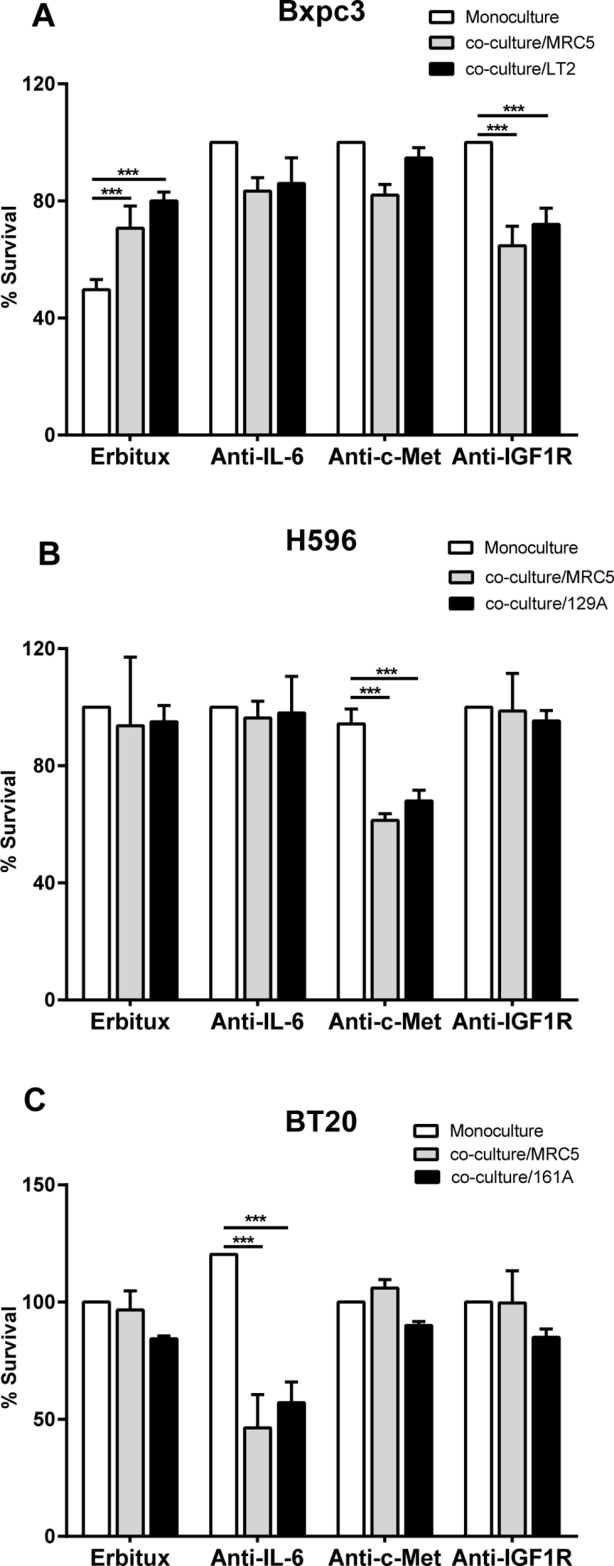
Co-culturing the tumor cells with fibroblasts influences their response to therapeutic agents. Cancer cells and fibroblasts (MRC5 or primary TAFs) were co-cultured as described in the cell viability assay for 5 days in the presence of inhibitory antibodies against EGFR, cMet, IL6 and or IGF1R. Cell viability was measured on day 5 using the CellTiterGlo as described for the cell viability assay. The percentage of surviving cells (% survival) was calculated relative to the respective IgG control. **A)** The mono-cultured BxPc3 cells treated with Erbitux exhibited a significant reduction in cell survival, whereas in the BxPc3 cells co-cultured with MRC5 or LT2 cells were not strongly affected by Erbitux (approximately 20%). Upon treatment with the IGF1R antibody, the survival of Bxpc3 cells was moderately reduced in the co-cultures compared to the monocultures. Treatment with the IL6 and or cMet antibodies induced a slight reduction in cell survival. **B)** Upon treatment with the anti-cMet antibody lung cancer cell line, H596, did not exhibit a significant reduction in cell survival in monoculture. In co-culture with MRC5 or 129A TAFs, treatment with the anti-cMet antibody induced a significant reduction of in cell survival. **C)** The breast cancer cell line, BT20 responded to treatment with the anti-IL6 antibody in co-culture exhibiting a significant reduction of cell survival. The mono-cultured BT20 cells treated with the anti- IL6 antibody exhibited no reduction in cell survival.

The pancreatic cancer cells (Bxpc3), in monoculture were sensitive (approximately 50% survival) to treatment with Erbitux. However, in co-culture with either MRC5 cells or the pancreatic fibroblasts (LT2), these cells were less sensitive or were partially resistant (approximately 75% survival) to the same treatment. Additionally, Bxpc3 in co-culture responded to mAb IGF1R treatment (approximately 30% inhibition of proliferation) (Fig 6A).

The lung cancer cells (H596), exhibited no significant reduction in survival when treated with Erbitux, mAb IL6 or mAb IGF1R in monoculture. In co-culture with MRC5 cells or primary lung TAFs (129A) a significant reduction in survival (approximately 40%) was observed upon treatment with mAb cMet (Fig 6B), but not with the other therapeutic agents used.

The survival of the breast cancer cells (BT20) in co-culture with MRC5 cells was significantly reduced (approximately 50%) upon treatment with by mAb IL6. This effect was not observed when the BT20 cells were in monoculture (Fig 6C). Treatment with Erbitux, mAb cMet or mAb IGF1R did not influence the survival of BT20 cells in monoculture or co-culture with corresponding fibroblasts.

## Discussion

Although it is evident that tumor-stroma crosstalk appears to play a critical role in tumor progression, and resistance to therapeutic agents, few suitable *in vitro* tools/models are available to examine these interactions. Most of the *in vitro* data regarding the efficacy of therapeutic agents have been obtained from 2D mono-cultures of cancer cells in which the stromal component is lacking or from trans-well systems in which the tumor cells and stromal cells are physically separated. Alternatively, *in vivo* data have been obtained from xenograft models in which human tumor cells interact with mouse stromal cells. However, this microenvironment, if at all, is a poor substitute for the human TME. These *in vitro* and *in vivo* methods may overestimate the effects of therapeutic agents, in contrast to co-culture models in which human tumors cells and fibroblasts of human origin directly interact with each other. The co-culture model we described in this study involves culturing tumor cells and fibroblasts in a 3D setting that mimics the *in vivo* micro-environment. This model enables the monitoring of the effects of co- culturing and the contribution of the crosstalk between tumor cells and fibroblasts *in vitro* in the absence of exogenous factors, such as serum, growth factors or hormones, on cell survival. Our data from the experiment comparing trans-well based co-cultures and 2D co-cultures to 3D co-culture model clearly indicated that 3D co-culture exerts a differential impact on cell survival. Using this model, we revealed for the first time that different cancer cell types elicit distinct sets of secreted factors from stromal fibroblasts and, thus, can uniquely influence cell survival and therapeutic responses to therapeutic agents.

We used cancer cells from different tumor types and FAP-positive fibroblasts (S1 Fig) from different origins, including primary TAFs, for the co-culture experiments. Upon dissociation of spheroids on day 5 to identify the proliferating population, we found that the predominant proportion of the proliferating cells in the co-cultures was cancer cells (EpCAM-positive) (S2 Fig). However, co- culturing with fibroblasts did not induce enhanced proliferation of all cancer cell lines tested. In fact, there were some cell lines that proliferated either equally well or better as mono-cultures indicating that there may be other factors influencing cell survival in co-cultures. The interaction between tumor cells and fibroblasts has been reported to induce the secretion of a variety of growth factors and cytokines by fibroblasts or cancer cells [18]. The expression of various soluble factors is associated with poor prognosis and may be of predictive value. It is known that the growth factor /cytokine (GC) profiles of cancer patients vary depending on the cancer type and stage. In accordance with this understanding, the GC profile of our co-culture models varied depending on the cancer type. The GC profile of mono-cultured fibroblasts showed that although certain soluble factors were commonly produced by all fibroblasts, clear differences were observed between fibroblasts from different cancer types. For example IL-6 and IL-8 were secreted by all the fibroblasts we tested whereas HGF was specifically secreted by lung fibroblasts (S3 Fig). Further, we observed that EGF is primarily secreted by co-cultures of pancreatic and breast cancer cells whereas HGF is primarily secreted by lung cancer cells and fibroblasts, and that IL6 is primarily secreted by the breast cancer co-cultures (Fig 5) indicating a cancer specific pattern in cytokine secretion. However, we also observed that some cell lines, such as Suit2 and H1993, both of which did not exhibit increased survival upon co-culture with fibroblasts, secreted growth factors (PDGF and TGF α, respectively), already in monoculture (S6 Fig). It would be interesting to evaluate whether these growth factors play a key role in attributing this fibroblast independent cell survival in this setting.

Our results from the pancreatic cancer cell panel indicated that most of the cell lines depended on fibroblasts for survival or are at least interdependent under these conditions. These data reflect the clinical situation, in which a desmoplastic stromal reaction containing fibroblasts is considered as a hallmark of pancreatic cancer [5, 12]. Pancreatic cancer cells are known to depend on EGFR signaling, and therapies targeting this signaling pathway are under evaluation in the clinic. However, the efficacy of drugs targeting EGFR is limited [17]. The pancreatic cancer cell line, Bxpc3, exhibited reduced sensitivity to Erbitux in co-culture compared to mono-culture. One reason for this change in the response to Erbitux treatment could be the differential expression of EGFR between the mono-culture and the co-culture. However, we did not detect a significant difference in the EGFR levels between the mono- and co-cultures (S5A Fig), indicating that the resistance of these cells to treatment with Erbitux occurs *de novo* and is potentially mediated by co-culturing with fibroblasts. Considering the recent findings that have implicated a role of the IGF1R pathway and the EGFR in pancreatic cancer progression and therapeutic responses [16, 19], we treated the Bxpc3 cells with mAb IGF1R to determine whether the IGF1R influences the survival of these co-cultures In agreement with the clinical data, the Bxpc3 cells responded to IGF1R inhibition, suggesting that a combination therapy blocking the EGFR and IGF1R pathways may provide synergistic value in the clinic.

The resistance of lung cancer cell line, H596, to Erbitux in co-culture with fibroblasts and a corresponding increase in cMet expression and activation compared to the mono-cultures (S5B Fig), indicate that these cells have become resistant to EGFR therapy and depend on HGF produced by co-cultured fibroblasts for survival in co-cultures (Fig 6B). These results are in agreement with the data from other groups demonstrating that HGF produced by fibroblasts promotes tumor progression and induces resistance to EGFR inhibitors in lung cancer [20]. These observations further reflect the situation in non-small cell lung cancer patients, where treatment with inhibitors of the HGF pathway in combination with EGFR inhibitors has been suggested to serve as a better treatment strategy than treatment with either inhibitor alone[21].

The BT20 breast cancer cells used in these experiments were isolated from a breast tumor that was triple negative for the expression of Her2, estrogen receptor and progesterone receptor. Such triple negative tumors often express EGFR [22]. Triple negative breast cancer is also associated with increased stromal reaction with inflammatory component and a poor prognosis. In our co-culture model, BT20 cells secreted increased amounts of various inflammatory cytokines, including the pro-inflammatory cytokine IL6 (S4 Fig). IL6, has been reported to induce cell proliferation in various cancers [23]. We also observed the activation of STAT3, a transcription factor that is downstream of the IL6 receptor, in the co-cultured BT20 cells, indicating the activation of this pathway (S5C Fig). Blocking IL6 resulted in a significant decrease in the survival of BT20 cells that were co-cultured with fibroblasts, indicating that the activation of STAT3 and the subsequent increase in cell proliferation are mediated by IL6. Importantly, the BT20 co-cultures also secreted EGF but did not respond to Erbitux treatment. In this context, it is important to note that the activation of the IL6 pathway has been implicated in the resistance to Trastuzumab (anti-Her2) in PTEN-deficient tumor cells [24]. Thus, IL6 secretion in BT20 co-cultures may be responsible for the resistance to Erbitux in our model. These data suggest that tumor cells can acquire resistance to therapeutic agents by utilizing alternative pathways depending on the availability of the corresponding ligands and the composition of the TME.

Considering the complexity and the dynamics of tumor cell—fibroblast interactions in the TME *in vivo*, our 3D co-culture system has also potential limitations. The ratio of tumor cells to fibroblasts and the time point of the measurements, for e.g. may need to be optimized based on the indication and the cell type examined. Other factors, such as mutations in certain genes that influence proliferation, were not taken into account in our system and may also contribute to the differential survival of the co-cultures with fibroblast. Nevertheless, this co-culture model can be used as a tool to elucidate the efficacy of potential therapies and/or the mechanisms underlying the resistance to these therapies *in vitro*. This 3D co-culture system can be reliably used as a method for *in vitro* pre-clinical studies to understand tumor-stroma interactions. Furthermore, the use of patient-derived primary cells could further increase the predictive value of this method. The possibility to extend this system to other cells of in the TME, including immune cells, is very attractive, and this advancement will be of great value once established.

## Supporting Information

S1 Fig The expression of fibroblast activation protein (FAP) by MRC5 and LT2 fibroblasts and primary TAFs. The cell surface expression of FAP, a fibroblast activation marker, was measured on fibroblast cells (MRC5 and LT2) and primary TAFs (129A and 161A) via flow cytometry. We observed that all the fibroblasts used expressed FAP on their cell surface.
(TIF)

S2 Fig Tumor cell fibroblast co-culture induces cell proliferation and spheroid formation. Cells were cultured either in monoculture or co-culture as indicated for the cell viability assay. Phase contrast images of mono and co-cultures were taken on day 5 using an inverted microscope with 20x magnification. All the cell lines investigated showed no or minimal formation of spheroids in monoculture. Upon co-culture with the MRC5 cells all three cell lines formed multicellular spheroids by day 5. Confocal imaging was performed on day 5 as described in M&M section with pre-labeled tumor cells and fibroblasts. The distribution of fibroblasts in spheroids varied between cell lines. The Bxpc3 and BT20 cells formed tight spheroids and the fibroblasts were mostly outside the spheroid unlike H596 which formed loose spheroids the fibroblasts were found within the spheroid as well. FACS analysis of cell populations in co-culture spheroids was performed on day 5. Cells were cultured as indicated earlier. Spheroids were collected and treated with cell dissociation reagent to get single cells for the analysis. Cell suspensions were incubated with anti-FAP antibody (activated fibroblast/ marker) or with anti-EpCAM antibody (Epithelial cell marker). Tumor cells expressed EpCAM and could be detected in monoculture as well as co-culture with all the cell lines. However, few or no fibroblasts could be detected on day 5 indicating that even though initially more fibroblasts were added than tumor cells, the co-culture conditions favored tumor cell proliferation.
(TIF)

S3 Fig GC profiles of the MRC5 and LT2 fibroblasts and the primary TAFs. The supernatants from mono-cultured fibroblast spheroids were collected on day 5, and 42 different growth factors and cytokines were measured using Luminex multiplex technology. The growth factors and cytokines that were produced at detectable levels are depicted in the graph. Among these growth factors, the lung fibroblast cell lines MRC5 and 129A produced higher levels of HGF and VEGF than the pancreatic fibroblast cell line LT2 and the primary breast TAF cell line 161A. The LT2 cells secreted higher levels of PDGF than the other fibroblast cell types. Regarding the cytokines, all of the fibroblasts secreted high levels of IL6 and IL8. The expression of MCSF was higher in 161A breast TAFs than in the other fibroblast cell types. The LT2 pancreatic fibroblasts produced higher levels of G-CSF and GM-CSF than the other fibroblast cell types.
(TIF)

S4 Fig GC profiles of the tumor cell-MRC5 fibroblast co-cultures. The supernatants from co-culture spheroids were collected on day 5, and 42 different growth factors and cytokines were measured using Luminex multiplex technology. The growth factors and cytokines that were produced at detectable levels are depicted in the graph.
(TIF)

S5 Fig Differential expression and activation of EGFR, cMet and STAT3 in the 3D co-cultures. Cancer cells and fibroblasts (MRC5) were cultured as either monocultures or co-cultures for 5 days as described in the cell viability assay. On day 5, spheroids were collected, and lysates were prepared for Western blot. **A.** The expression of EGFR and phospho-EGFR, the activated form of EGFR, was detected in the Bxpc3 lysates using specific antibodies. Although the EGFR levels were only slightly increased in the co-cultures with the MRC5 cells, the expression of the phosphorylated form of EGFR was clearly increased in the co-cultures compared to the monocultures. **B.** The expression of cMet was detectable In H596 cells that were monocultured as well as those that were co-cultured with MRC5 cells. However, the cMet expression level was higher in the co-cultures, and the expression of phospho-cMet was only detected in the co-cultures. **C.** Although the monocultured BT20 cells expressed STAT3, they did not exhibit the activation of this factor. The level of p-STAT3 was increased in the co-cultured BT20 cells and was also detectable in the monocultured fibroblasts.
(TIF)

S6 Fig Growth factor secretion by cell lines that were not dependent on fibroblast co-culture for survival. The supernatants from co-culture spheroids of cell lines that were not dependent on fibroblast co-culture for survival were collected on day 5, and 42 different growth factors and cytokines were measured using Luminex multiplex technology. The growth factors and cytokines that were produced at detectable levels are depicted in the graph.
(TIF)
